# Assessing frailty to predict surgical risk: a comparative study of three tools in older non-cardiac surgery patients

**DOI:** 10.1186/s12877-025-06683-1

**Published:** 2025-11-21

**Authors:** Mantana Saetang, Thitikan Kunapaisal, Sunisa Chatmongkolchart, Dararat Yongsata, Khwanrut Sukitpaneenit

**Affiliations:** Department of Anesthesiology, Faculty of Medicine, Prince of Songkla University, Hat-Yai, Songkhla, Thailand

**Keywords:** Older adult, Frailty, CFS, MFI-11, FRAIL scale, Postoperative complications.

## Abstract

**Background:**

Frailty is a significant predictor of adverse outcomes in older surgical patients. this study, we aimed to evaluate the feasibility and predictive ability of the Clinical Frailty Scale (CFS), Modified Frailty Index-11 (mFI-11), and FRAIL scale for postoperative complications in older Thai patients who underwent intermediate- to high-risk non-cardiac surgery.

**Methods:**

This prospective cohort study included 637 older patients (aged ≥ 60 years) scheduled for intermediate- to high-risk elective non-cardiac surgery. Frailty was assessed preoperatively using the CFS, mFI-11, and FRAIL scale. Postoperative complications were defined as Clavien–Dindo classification ≥ 2. Predictive performance was analyzed using logistic regression and the area under the receiver operating characteristic curve (AUC).

**Results:**

The mean age of participants was 70.5 years (standard deviation 7.68), and 48% were male. Frailty was significantly associated with higher rates of postoperative complications across all tools: CFS (44.9% vs. 22.2%), mFI-11 (57.8% vs. 26.9%), and FRAIL scale (56.3% vs. 26.0%) (all *p* < 0.001). In multivariable logistic regression, the CFS was the only independent predictor (odds ratio 2.39, 95% confidence interval [CI]: 1.42–4.00, *p* < 0.001). Area under the curve (AUC) values were 0.635 (95% CI: 0.5902–0.6794) (mFI-11), 0.632 (95% CI: 0.5881–0.6756) (FRAIL scale), and 0.619 (95% CI: 0.5742–0.6637) (CFS), compared with 0.657 (95% CI: 0.6152–0.6988) of the American Society of Anesthesiologists (ASA) classification. Combining frailty tools with ASA improved predictive accuracy, with CFS + ASA exhibiting the highest AUC (0.704, 95% CI: 0.660–0.748).

**Conclusion:**

Frailty assessed with the CFS, mFI-11, and FRAIL scale was associated with postoperative complications, with the CFS demonstrating the strongest independent predictive value. Incorporating frailty screening into preoperative evaluation, especially combined with ASA classification, can improve risk stratification and perioperative care.

**Trial registration:**

TCTR20210706002.

## Background

 Population aging is accelerating globally. As healthcare and living conditions improve, life expectancy continues to rise, resulting in a growing proportion of older adults. Thailand is among the most rapidly aging countries in Asia, transitioning from a young to an aging society within only a few decades. By 2050, the number of older adults in Thailand is projected to reach 20 million, accounting for 35.8% of the total population [1–3]. As the aging population continues to expand, the number of surgical procedures performed in this group is also expected to increase substantially [4].

One major challenge in this context is frailty, a multidimensional geriatric syndrome in older adults characterized by reduced physical strength and physiological reserve, which increases vulnerability to falls, illness, and other health complications [5]. More than half of older surgical patients are estimated to experience preoperative frailty [6]. Furthermore, frailty is strongly associated with surgical complications, mortality, and prolonged length of stay (LOS), significantly challenging healthcare providers and the health system [7–12].

Risk prediction models, such as age, American Society of Anesthesiologists (ASA) physical status, Revised Lee’s Cardiac Risk Index, Charlson Comorbidity Index, and exercise tolerance, are commonly used to estimate perioperative risk. Nonetheless, these tools mainly predict organ-specific complications and do not account for the frailty-associated diminished reserve and vulnerability, thereby reducing their ability to capture outcomes, including functional recovery, LOS, and postoperative institutionalization [13]. Therefore, incorporating frailty and geriatric syndrome assessment into the preoperative evaluation is essential for improving the prediction of adverse outcomes.

Several frailty assessment tools are available. The most widely used are the Fried Criteria and Rockwood Frailty Index [5, 14]. While valuable, they are often time-consuming and resource-intensive, limiting their feasibility in routine clinical practice. Consequently, simplified screening tools are increasingly recognized as essential for the practical assessment of frailty in surgical patients. Nevertheless, a consensus on which measures are most appropriate for perioperative settings is lacking [15]. Ideally, a frailty assessment tool should be quick, easy to administer, and effective in predicting perioperative risk and guiding care decisions [16]. Recently developed tools, such as the CFS, mFI-11, and FRAIL scale, can be administered in approximately 10 min without requiring physical measurement such as hand grip strength [17]. Despite their growing use, systemic evaluation of their validity, feasibility, and predictive value for postoperative complications remains warranted.

Therefore, in this study, we aimed to evaluate the content validity and feasibility of the CFS, mFI-11, and FRAIL scale, to compare their frailty prevalence estimates, and to assess their predictive ability for postoperative complications (Clavien–Dindo classification ≥ 2) in older adults undergoing intermediate- to high-risk non-cardiac surgery.

## Methods

### Ethical statements

This study was conducted in accordance with the principles of the Declaration of Helsinki and was reviewed and approved by the Institutional Ethics Committee of the Faculty of Medicine, Prince of Songkla University, Songkhla, Thailand (Approval Reference: REC.64–396-8-1) on February 27, 2022. All the enrolled patients provided informed consent to participate in the study.

### Study design, setting, and participants

**Fig. 1:**
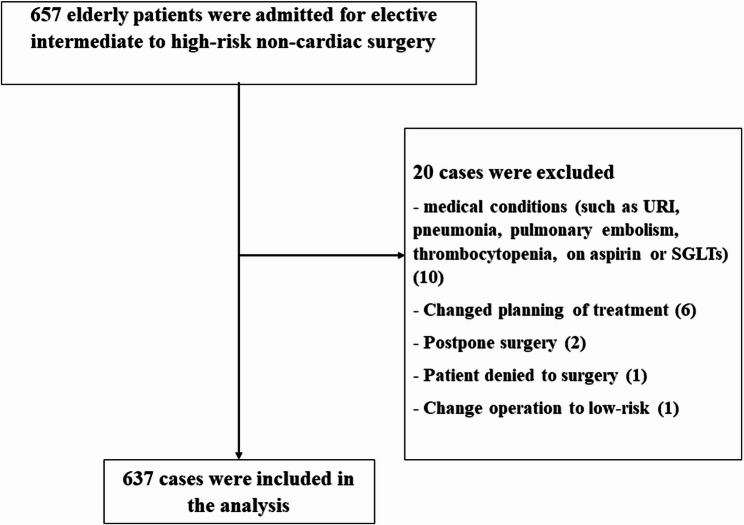
Consort flow of study

This prospective cohort observational study was conducted among older patients undergoing scheduled non-cardiac surgery at Songklanagarind Hospital, a tertiary care center in Southern Thailand between January 30, 2023 and April 10, 2024. The study included patients aged ≥ 60 years who were scheduled for elective non-cardiac surgery. Patients undergoing surgery categorized as moderate-to-high risk based on the cardiac risk stratification for non-cardiac procedures were eligible for inclusion. Only the first surgery during hospitalization was considered for each patient. Patients who were too ill for preoperative assessments and those receiving palliative care were excluded. In total, 657 patients were initially admitted for surgery, of whom 20 were excluded from the analysis. Thus, 637 patients were included in the final analysis (Figure 1).

### Sample size calculation

Our primary objective was to compare the predictive performance (receiver operating characteristic [ROC]–area under the curve [AUC]) of three brief frailty tools (CFS, mFI-11, and FRAIL scale) for in-hospital postoperative complications ≥ grade II by Clavien–Dindo. Target AUCs were obtained from prior studies:[18, 19] 0.684 (95% CI 0.614–0.749) [18], 0.753 (95% CI 0.686–0.812) [18], and 0.60 (95% CI 0.58–0.62) for the FRAIL scale, CFS, and mFI, respectively [19]. Using a two-sided α = 0.05 (Zα/2 = 1.96) and power of 80% (Zβ = 0.84), while assuming equal allocation (r = 1), we estimated the sample sizes required for pairwise AUC comparisons by approximating the AUC as a probability and applying a z-test for the difference between two independent proportions with continuity correction). These proportions were used to calculate the required sample size for the analysis as follows:

$$\:{n}_{1}={\left[\frac{{z}_{1-\frac{\alpha\:}{2}}\sqrt{\stackrel{-}{p}\stackrel{-}{q}\left(1+\frac{1}{r}\right)}+{z}_{1-\beta\:}\sqrt{{p}_{1}{q}_{1}+\frac{{p}_{2}{q}_{2}}{r}}}{{\Delta\:}}\right]}^{2}$$

$$\:r=\frac{{n}_{2}}{{n}_{1}},\hspace{1em}{q}_{1}=1-{p}_{1},\hspace{1em}{q}_{2}=1-{p}_{2}$$

$$\:\stackrel{-}{p}=\frac{{p}_{1}+{p}_{2}r}{1+r},\hspace{1em}\stackrel{-}{q}=1-\stackrel{-}{p}$$

- CFS vs. FRAIL scale: n₁ = n₂ = 637 per group (largest requirement)
- CFS vs. mFI-11: n₁ = n₂ = 159 per group
- FRAIL vs. mFI-11: n₁ = n₂ = 534 per group

Because all three tools were applied to the same cohort (within-subject comparisons), we adopted the most conservative requirement and planned for N = 637 participants to ensure ≥80% power across all pairwise comparisons. We ultimately analyzed 637 patients, meeting the a priori target.

### Data collection

#### Frailty assessment tools

Preoperative frailty assessments were performed using the CFS, mFI, and FRAIL scale. The CFS provides a clinical approach to frailty. It comprises nine clinical stages, with scores of 1 to 2 indicating robustness, 3 or 4 representing prefrailty, and ≥5 identifying frailty [20]. The mFI-11 is a scoring index of 11 accumulating deficits that contribute to frailty. Frailty status is categorized as robust (score = 0), prefrail (< 0.27), and frail (≥ 0.27) [21]. The FRAIL scale is a validated phenotypic tool comprising five questions related to fatigue, resistance, ambulation, illness, and weight loss. A score of 0 indicates robustness, 1–2 represents pre-frailty, and ≥3 identifies frailty [22–24]. 

Preoperative data retrieved from medical records included age, gender, marital status, living situation, presence of comorbidities, Mini-Cog score, medications used on admission, ASA score, mid-arm circumference, albumin level, hemoglobin level, visual impairment, hearing impairment, surgical subspecialty, and cardiac risk stratification. Regarding cognitive screening, the Mini-Cog test (Thai version) was used, with a score < 3 indicating a higher likelihood of cognitive impairment [25]. 

For laboratory markers, preoperative hemoglobin, albumin, and serum creatinine levels were obtained as part of routine clinical care; they were selected because they reflect physiological reserve and have been associated with frailty and adverse surgical outcomes in prior studies [26–28]. Low hemoglobin may indicate anemia; hypoalbuminemia is a marker of malnutrition and chronic illness; and elevated serum creatinine reflects impaired renal function. Therefore, we examined their associations with frailty status and short-term postoperative outcomes in this cohort.

#### Postoperative complications

Postoperative complications were systematically monitored and categorized as follows: cardiac, pulmonary, neurological, and renal complications; infections; multi-organ failure; transfusions; unplanned returns to the operating room or intensive care unit; LOS; 30-day readmission; and all-cause mortality. Postoperative complications were categorized according to the Clavien–Dindo classification [29].

#### Outcomes

The sensitivity, specificity, ROC, and AUC of the three frailty scales in predicting in-hospital postoperative complications according to the Clavien–Dindo classification (≥ grade 2) were evaluated.

### Statistical analysis

Categorical variables were expressed as numbers and percentages, and differences between groups were compared using Chi-Square or Fisher’s exact test, as appropriate. Continuous variables were expressed as mean and standard deviation (SD) or median and interquartile range, and differences between groups were compared using a t-test or Wilcoxon rank sum test, as appropriate. 

Binary logistic regression analysis was conducted to assess the association between frailty (measured by the CFS, mFI-11, and FRAIL scale) and postoperative complications (Clavien–Dindo classification ≥ II). Univariable analyses were first conducted, and variables with p < 0.2 as well as clinically relevant covariates (age, sex, ASA classification, and major comorbidities) were included in the multivariable model. Backward stepwise elimination based on the Akaike Information Criterion was applied to identify independent predictors. Odds ratios (ORs) with 95% confidence intervals (CIs) were reported.

Model discrimination was evaluated using ROC curves, and the AUC was reported with 95% CIs. The discriminatory performance of the frailty instruments (CFS, mFI-11, and FRAIL) was compared using DeLong’s test for correlated ROC curves. All statistical tests were two-tailed, with p < 0.05 considered statistically significant. Analyses were performed using R software (version 4.3.1; R Foundation for Statistical Computing, Vienna, Austria).

## Results

### Patient characteristics by frailty assessment tool

We assessed 637 older Thai patients who underwent intermediate- to high-risk non-cardiac surgery (Figure 1). Of these, 464 (72.8%) underwent intermediate-risk non-cardiac surgery. The mean patient age was 70.5 years (SD 7.68), and males accounted for 48% of the patients. According to the CFS, mFI-11, and FRAIL scale, the prevalence rates of frailty in the study population were 38.5%, 13%, and 16.2%, respectively. Patient characteristics were analyzed according to the CFS, mFI-11, and FRAIL scale. Frail patients had a significantly higher prevalence of preoperative anemia and lower albumin levels, creatinine clearance, and Mini-Cog scores than prefrail and robust patients (Table 1).

**Table 1:** Patient characteristics by frailty measure in elderly patients undergoing intermediate to high-risk non-cardiac surgery

Patient characteristics	CFS				mFI-11				Frail scale				
	robust (*n* = 38)	prefrail (*n* = 354)	frail (*n* = 245)	*p*-value	robust (*N* = 225)	prefrail (*N* = 329)	frail (*N* = 83)	*p*-value		robust (*N* = 182)	prefrail (*N* = 352)	frail (*N* = 103)	*p*-value
Age (yrs), median(IQR)	67 (62.2,70.8)	68 (64,73)	73 (67,79)	< 0.001	68 (64,73)	70 (65,75)	74 (69,83)	< 0.001	66.5 (63,72)		70 (65,76)	73 (68,78)	< 0.001
Sex; male, n (%)	17 (44.7)	194 (55.1)	95 (38.8)	< 0.001	112 (50)	163 (49.7)	31 (37.3)	0.105	84 (46.7)		161 (45.7)	61 (59.2)	0.049
BMI, median(IQR)	25.6 (21.6,28.7)	24 (21.6,27.2)	23.4 (20.4,26.6)	0.014	24.2 (21.8,27.2)	23.8 (20.9,27)	23.4 (20.7,26.7)	0.165	23.8 (21.3,26.6)		24.1 (21.6,27.6)	23.2 (20.3,26.3)	0.014
Marital status, n (%)				0.001				0.007					0.091
- married/common law	30 (78.9)	281 (79.6)	161 (66)		180 (80)	238 (72.8)	54 (65.1)		151 (83)		247 (70.4)	74 (72.5)	
- never married	3 (7.9)	15 (4.2)	8 (3.3)		7 (3.1)	18 (5.5)	1 (1.2)		6 (3.3)		16 (4.6)	4 (3.9)	
- widowed	3 (7.9)	41 (11.6)	58 (23.8)		31 (13.8)	48 (14.7)	23 (27.7)		19 (10.4)		64 (18.2)	19 (18.6)	
- divorced/separated	2 (5.3)	16 (4.5)	17 (7)		7 (3.1)	23 (7)	5 (6)		6 (3.3)		24 (6.8)	5 (4.9)	
Education, n (%)				< 0.001				0.058					0.001
- less than high school	13 (34.2)	165 (46.6)	168 (68.9)		109 (48.4)	183 (55.6)	54 (65.9)		81 (44.5)		203 (57.8)	62 (60.2)	
- high school	9 (23.7)	92 (26)	46 (18.9)		54 (24)	78 (23.7)	15 (18.3)		41 (22.5)		80 (22.8)	26 (25.2)	
- ≥ bachelor’s degrees	16 (42.1)	97 (27.4)	30 (12.3)		62 (27.6)	68 (20.7)	13 (15.9)		60 (33)		68 (19.4)	15 (14.6)	
Living situation, n (%)				< 0.001				< 0.001					0.001
- living at home independently	10 (26.3)	128 (36.2)	184 (75.1)		94 (41.8)	172 (52.3)	56 (67.5)		83 (45.6)		182 (51.7)	57 (55.3)	
- living at home with help	1 (2.6)	4 (1.1)	31 (12.7)		1 (0.4)	25 (7.6)	10 (12)		4 (2.2)		20 (5.7)	12 (11.7)	
- lives alone	27 (71.1)	222 (62.7)	30 (12.2)		130 (57.8)	132 (40.1)	17 (20.5)		95 (52.2)		150 (42.6)	34 (33)	
Comorbidities, n (%)	32 (84.2)	332 (93.8)	232 (94.7)	0.048	209 (92.9)	307 (93.3)	80 (96.4)	0.522	157 (86.3)		339 (96.3)	100 (97.1)	< 0.001
- Ischemic heart disease	1 (3.1)	34 (10.1)	28 (12.1)	0.285	6 (2.9)	35 (11.3)	22 (27.5)	< 0.001	3 (1.9)		19 (5.6)	41 (41)	< 0.001
- Heart failure	0 (0)	6 (1.8)	4 (1.7)	1	1 (0.5)	4 (1.3)	5 (6.2)	0.008	0 (0)		6 (1.8)	4 (4)	0.047
- Hypertension	7 (21.9)	214 (63.9)	173 (74.6)	< 0.001	104 (49.8)	226 (72.9)	64 (80)	< 0.001	36 (22.5)		269 (79.4)	89 (89)	< 0.001
- Dyslipidemia	10 (31.2)	212 (63.5)	152 (65.5)	< 0.001	115 (55)	197 (63.8)	62 (77.5)	0.002	72 (45.3)		229 (67.6)	73 (73)	< 0.001
- Diabetes	1 (3.1)	73 (21.9)	54 (23.3)	0.032	20 (9.6)	75 (24.4)	33 (41.2)	< 0.001	7 (4.4)		76 (22.4)	45 (45)	< 0.001
- Chronic lung disease	1 (3.1)	19 (5.7)	19 (8.2)	0.367	4 (1.9)	21 (6.8)	14 (17.5)	< 0.001	3 (1.9)		23 (6.8)	13 (13)	0.002
- CKD stage more than or equal to 3	1 (3.1)	40 (12)	31 (13.4)	0.25	13 (6.2)	47 (15.3)	12 (15)	0.005	10 (6.4)		38 (11.2)	24 (24)	< 0.001
- CVD	2 (6.2)	24 (7.2)	30 (12.9)	0.061	9 (4.3)	37 (12.1)	10 (12.5)	0.007	3 (1.9)		30 (8.8)	23 (23)	< 0.001
- Alcohol drinking	6 (18.8)	61 (18.4)	30 (12.9)	0.21	36 (17.2)	53 (17.3)	8 (10)	0.263	29 (18.5)		49 (14.5)	19 (19)	0.382
- Smoking	5 (15.6)	67 (20.2)	36 (15.5)	0.342	41 (19.6)	56 (18.2)	11 (13.8)	0.51	30 (19.1)		58 (17.1)	20 (20)	0.75
Visual impairment, n (%)	17 (44.7)	227 (64.1)	142 (58)	0.038	126 (56)	206 (62.6)	54 (65.1)	0.197	108 (59.3)		212 (60.2)	66 (64.1)	0.718
Hearing impairment, n (%)	7 (18.4)	120 (33.9)	79 (32.2)	0.153	62 (27.6)	116 (35.3)	28 (33.7)	0.157	50 (27.5)		116 (33)	40 (38.8)	0.134
ASA class, median(IQR)	2 (2,2)	2 (2,3)	3 (2,3)	< 0.001	2 (2,3)	3 (2,3)	3 (2,3)	< 0.001	2 (2,2)		2 (2,3)	3 (3,3)	< 0.001
RCRI, n (%)				0.579				< 0.001					< 0.001
− 0	29 (76.3)	208 (58.8)	150 (61.2)		161 (71.6)	191 (58.1)	35 (42.2)		136 (74.7)		221 (62.8)	30 (29.1)	
− 1	8 (21.1)	120 (33.9)	75 (30.6)		61 (27.1)	107 (32.5)	35 (42.2)		42 (23.1)		114 (32.4)	47 (45.6)	
− 2	1 (2.6)	22 (6.2)	16 (6.5)		3 (1.3)	31 (9.4)	13 (15.7)		4 (2.2)		17 (4.8)	26 (25.2)	
Cardiac risk stratification surgical procedure, n (%)				0.021				0.21					0.655
- High-risk (172)	9 (23.7)	111 (31.4)	52 (21.3)		51 (22.9)	96 (29.2)	25 (30.1)		45 (24.7)		97 (27.6)	30 (29.4)	
- Intermediate-risk (463)	29 (76.3)	242 (68.6)	192 (78.7)		172 (77.1)	233 (70.8)	58 (69.9)		137 (75.3)		254 (72.4)	72 (70.6)	
Preoperativ anemia, n (%)	17 (44.7)	188 (53.1)	180 (73.5)	< 0.001	114 (50.7)	209 (63.5)	62 (74.7)	< 0.001	93 (51.1)		216 (61.4)	76 (73.8)	< 0.001
Albumin level, median(IQR)	4 (3.7,4.3)	4 (3.8,4.3)	3.8 (3.5,4.1)	< 0.001	4.1 (3.9,4.3)	3.9 (3.6,4.1)	3.6 (3.2,4)	< 0.001	4 (3.8,4.3)		4 (3.6,4.2)	3.8 (3.4,4)	< 0.001
CrCl, median(IQR)	71.8 (62,80.8)	62.7 (49,77)	58 (43.4,76)	0.004	64 (52,81)	61 (45,76)	62 (43.2,75.6)	0.042	65.2 (54,80)		63 (46,77)	53 (39.3,71.8)	< 0.001
Mini-Cog score, median(IQR)	2 (1,3)	3 (1,3)	2 (1,3)	0.002	3 (1,3)	2 (1,3)	2 (0,3)	< 0.001	3 (1,3)		2 (1,3)	2 (1,3)	0.096

*ASA* American Society of Anesthesiologists, *BMI* Body Mass Index, *CKD* Chronic kidney disease, *CrCl* Creatinine clearance, *CVD* Cerebrovascular disease, *RCRI* Revised Cardiac Risk Index

### Association between frailty and postoperative complications by frailty assessment tool

Frailty assessed using the three tools was significantly associated with increased rates of overall postoperative complications. Specifically, frail patients demonstrated markedly higher complication rates than non-frail patients across all three assessment tools: CFS (44.9% vs. 22.2%), mFI-11 (57.8% vs. 26.9%), and FRAIL scale (56.3% vs. 26.0%) (all p < 0.001) (Table 2). Regarding specific outcomes, the proportion of frail patients with complications classified as Clavien–Dindo grade ≥ 2, indicating the presence of more severe postoperative complications, was higher than that of non-frail patients. Moreover, LOS was markedly prolonged in frail patients across all frailty assessment tools (Table 2).

**Table 2:** Post-operative complications and outcomes by frailty measure in elderly patients undergoing intermediate to high-risk non-cardiac surgery

Post-operative complications and Outcomes	CSF			mFI-11			FRAIL scale		
	Non-frail	Frail	*p*-value	Non-frail	Frail	*p*-value	Non-frail	Frail	*p*-value
Overall post-op complications, n (%)	87 (22.2)	110 (44.9)	< 0.001	149 (26.9)	48 (57.8)	< 0.001	139 (26)	58 (56.3)	< 0.001
Cardiac complication, n (%)	25 (27.8)	16 (14.5)	0.033	32 (21.1)	9 (18.8)	0.889	26 (18.4)	15 (25.4)	0.356
Pulmonary complication, n (%)	36 (40)	31 (28.2)	0.107	52 (34.2)	15 (31.2)	0.839	52 (36.9)	15 (25.4)	0.161
Neurologic complication, n (%)	6 (6.7)	15 (13.6)	0.18	13 (8.6)	8 (16.7)	0.189	15 (10.7)	6 (10.2)	1
Renal complication, n (%)	18 (20.2)	21 (19.1)	0.983	31 (20.5)	8 (16.7)	0.705	27 (19.3)	12 (20.3)	1
Infection, n (%)	16 (18)	25 (22.7)	0.517	32 (21.2)	9 (18.8)	0.873	33 (23.6)	8 (13.6)	0.161
Multiorgan failure, n (%)	1 (1.1)	1 (0.9)	1	1 (0.7)	1 (2.1)	0.425	1 (0.7)	1 (1.7)	0.506
Transfusion of blood products, n (%)	26 (29.2)	64 (58.2)	< 0.001	62 (41.1)	28 (58.3)	0.054	58 (41.4)	32 (54.2)	0.133
Use Vasopressor or inotrope, n (%)	4 (4.5)	9 (8.2)	0.448	12 (7.9)	1 (2.1)	0.195	10 (7.1)	3 (5.1)	0.759
Unplanned return to OR, n (%)	3 (3.4)	15 (13.6)	0.024	13 (8.6)	5 (10.4)	0.773	10 (7.1)	8 (13.6)	0.242
Unplanned return to ICU, n (%)	5 (5.6)	5 (4.5)	0.755	9 (6)	1 (2.1)	0.456	8 (5.7)	2 (3.4)	0.726
Duration of surgery (mins), median (IQR)	252.5 (175,370)	260 (180,350)	0.907	252.5 (180,360)	265 (182.5,342.5)	0.678	252.5 (175,355)	265 (190,360)	0.228
Blood loss (mL), median (IQR)	100 (20,300)	150 (50,400)	< 0.001	100 (20,300)	200 (40,425)	0.025	100 (20,300)	150 (50,475)	0.002
The Clavien-Dindo classification, median (IQR)	1 (1,1)	1 (1,3)	< 0.001	1 (1,1)	1 (1,3)	< 0.001	1 (1,1)	1 (1,3)	< 0.001
30-day mortality, n (%)	2 (0.5)	5 (2)	0.114	3 (0.5)	4 (4.8)	0.007	6 (1.1)	1 (1)	1
Death during admission, n (%)	1 (0.3)	3 (1.2)	0.161	2 (0.4)	2 (2.4)	0.084	3 (0.6)	1 (1)	0.507
Hospital LOS (day), median (IQR)	6 (4,9)	7 (5,11)	< 0.001	6 (4,9)	8 (6,12.5)	< 0.001	6 (4,9)	8 (6,11)	< 0.001
ICU LOS (day), media (IQR)	0 (0,0)	0 (0,0)	0.09	0 (0,0)	0 (0,0)	0.873	0 (0,0)	0 (0,0)	< 0.001
30-day readmission, n (%)	17 (4.3)	19 (7.8)	0.101	30 (5.4)	6 (7.2)	0.451	25 (4.7)	11 (10.7)	0.029

*ICU* Intensive care unit, *IQR* Interquartile range, *LOS* Length of stay, *OR* Operating room

Frailty assessed using the CFS significantly influenced various postoperative outcomes in older patients undergoing non-cardiac surgeries. Frail patients had a higher need for blood product transfusions and more frequent unplanned returns to the operating room than non-frail patients (58.2% vs. 29.2%, p < 0.001; 13.6% vs. 3.4%, p = 0.024, respectively). Despite these high complication rates, frail patients exhibited a lower incidence of cardiac complications than their non-frail counterparts (14.5% vs. 27.8%, p = 0.033).

Frailty assessment using the CFS, mFI-11, and FRAIL scale revealed varying associations with 30-day mortality rates among older surgical patients. There were no significant differences in 30-day mortality between non-frail and frail patients assessed using the CFS and FRAIL scale. However, the mFI-11 exhibited a substantial increase in mortality among frail patients than non-frail patients (3 [0.5%] vs. 4 [4.8%], p = 0.007). Frail patients demonstrated numerically higher rates of 30-day readmission across all frailty assessment tools; nonetheless, statistical significance was observed only for the FRAIL scale (p = 0.029) (Table 2).

### Frailty and postoperative complications according to the Clavien–Dindo classification

Among the 637 patients, 26.1% experienced postoperative complications. Using the CFS, mFI-11, and FRAIL scale, the incidences of complications in the frail and non-frail groups were 38% (93/245) and 18.6% (73/392), 49.4% (41/83) and 22.6% (125/554), and 45.6% (47/103) and 22.3% (119/534), respectively. The between-group differences were significant (p < 0.001) (Table 3).

**Table 3:** Factors associated with postoperative complications in elderly patients based on Clavien-Dindo classification (CDC)

Factor associated with postoperative complications	CDC < 2 (*N* = 471)	CDC class ≥ 2 (*N* = 166)	*p*-value
Age (yrs), median (IQR)	69 (65,74)	71.5 (65,78)	0.002
Sex;			< 0.001
Female	263 (56.1)	66 (39.8)	
Male	206 (43.9)	100 (60.2)	
BMI, median (IQR)	24.3 (21.6,27.6)	22.7 (20.2,25.3)	< 0.001
Living situation			< 0.001
living at home independently	227 (48.2)	95 (57.2)	
living at home with help	18 (3.8)	18 (10.8)	
lives alone	226 (48)	53 (31.9)	
Comorbidity	437 (92.8)	159 (95.8)	0.241
Ischemic heart disease	42 (8.9)	21 (12.7)	0.217
Heart failure	3 (0.6)	7 (4.2)	0.004
Hypertension	278 (59)	116 (69.9)	0.017
Dyslipidemia	268 (56.9)	106 (63.9)	0.141
Diabetes	83 (17.6)	45 (27.1)	0.012
Chronic lung disease	16 (3.4)	23 (13.9)	< 0.001
CKD stage ≥ 3	44 (9.3)	28 (16.9)	0.013
CVD (previous stroke or TIA)	38 (8.1)	18 (10.8)	0.354
Alcohol drinking	67 (15.3)	30 (18.9)	0.363
Smoking	67 (14.2)	41 (24.7)	0.003
Visual impairment	289 (61.4)	97 (58.4)	0.568
Hearing impairment	148 (31.4)	58 (34.9)	0.461
Hospital visit during a past year	403 (85.6)	142 (85.5)	1
Falling during a past year	80 (17)	28 (16.9)	1
ASA classification, median (IQR)	2 (2,3)	3 (2,3)	< 0.001
Revised Cardiac Risk Index			< 0.001
0	315 (66.9)	72 (43.4)	
1	130 (27.6)	73 (44)	
2	26 (5.5)	21 (12.7)	
Cardiac risk stratification surgical procedure			< 0.001
High-risk	106 (22.6)	66 (40)	
Intermediate-risk	364 (77.4)	99 (60)	
Preoperative anemia	260 (55.2)	125 (75.3)	< 0.001
Albumin, median (IQR)	4 (3.7,4.3)	3.8 (3.3,4)	< 0.001
CrCl, median (IQR)	64 (50,79.2)	55.7 (43,71.8)	< 0.001
Mini-Cog score, median (IQR)	2 (1,3)	2 (1,3)	0.1
Frail by CFS	152 (62)	93 (38)	< 0.001
Frail by mFI-11	42 (50.6)	41 (49.4)	< 0.001
Frail by FRAIL scale	56 (54.4)	47 (45.6)	< 0.001

*IQR* Interquartile range, *BMI* Body mass index, *CKD* Chronic kidney disease, *CVD* Cerebrovascular disease, *TIA* Transient ischemic attack, *ASA* American Society of Anesthesiologist

*CrCl* Creatinine clearance, *CFS* Clinical frail scale, *mFI-11* Modified frailty index-11

Univariate logistic analysis demonstrated that frailty was associated with postoperative complications regardless of which criteria were used: CFS (OR = 2.81, 95% CI: 1.93–4.09, p<0.001); mFI-11 (OR= 3.43, 95% CI: 2.11,5.58, p<0.001); and FRAIL scale (OR=3.01, 95% CI: 1.92,4.72, p<0.001) (Table 4). A multiple logistic regression analysis was performed to adjust for potential confounding variables. Only the CFS was found to be a significant predictor of postoperative complications (OR =2.39, 95% CI: 1.42–4, p<0.001) (Table 4).

**Table 4:** Univariate and multivariate analysis of factors associated with postoperative complications based on Clavien-Dindo classification (CDC)

Factor associated with CDC ≥ 2	Univariate		Multivariate	
	OR (95%CI)	*p*-value	OR (95%CI)	*p*-value
Sex: female vs. male	0.54 (0.37,0.79)	0.001	0.56 (0.32,0.97)	0.038
DM	1.73 (1.14,2.64)	0.011	1.56 (0.9,2.69)	0.115
CLD	4.51 (2.27,8.94)	< 0.001	2.02 (0.88,4.66)	0.098
CKD	1.82 (1.08,3.06)	0.023	0.56 (0.28,1.14)	0.111
ASA classification	3.84 (2.62,5.63)	< 0.001	1.48 (0.9,2.44)	0.118
CRSP: Intermediate-risk vs. High-risk	0.44 (0.3,0.65)	< 0.001	0.39 (0.22,0.7)	0.001
Albumin level	0.32 (0.22,0.46)	< 0.001	0.46 (0.3,0.73)	< 0.001
Frailty diagnosed by CFS	2.81 (1.93,4.09)	< 0.001	2.39 (1.42,4)	< 0.001
Frailty diagnosed by mFI-11	3.43 (2.11,5.58)	< 0.001	1.62 (0.85,3.09)	0.145
Frailty diagnosed by FRAIL scale	3.01 (1.92,4.72)	< 0.001	1.71 (0.96,3.07)	0.071

*ASA* American Society of anesthesiologist, *CDC* Clavien-Dindo classification, *CFS* Clinical frail scale, *CKD* Chronic kidney disease, *CLD* Chronic lung disease, *CRSP* Cardiac risk stratification surgical procedure, *DM* Diabetes mellitus, *mFI-11* Modified frailty index-11

### Comparison of the predictive ability of the ASA classification, frailty assessment scales, and their combination for postoperative complications

**Fig. 2:**
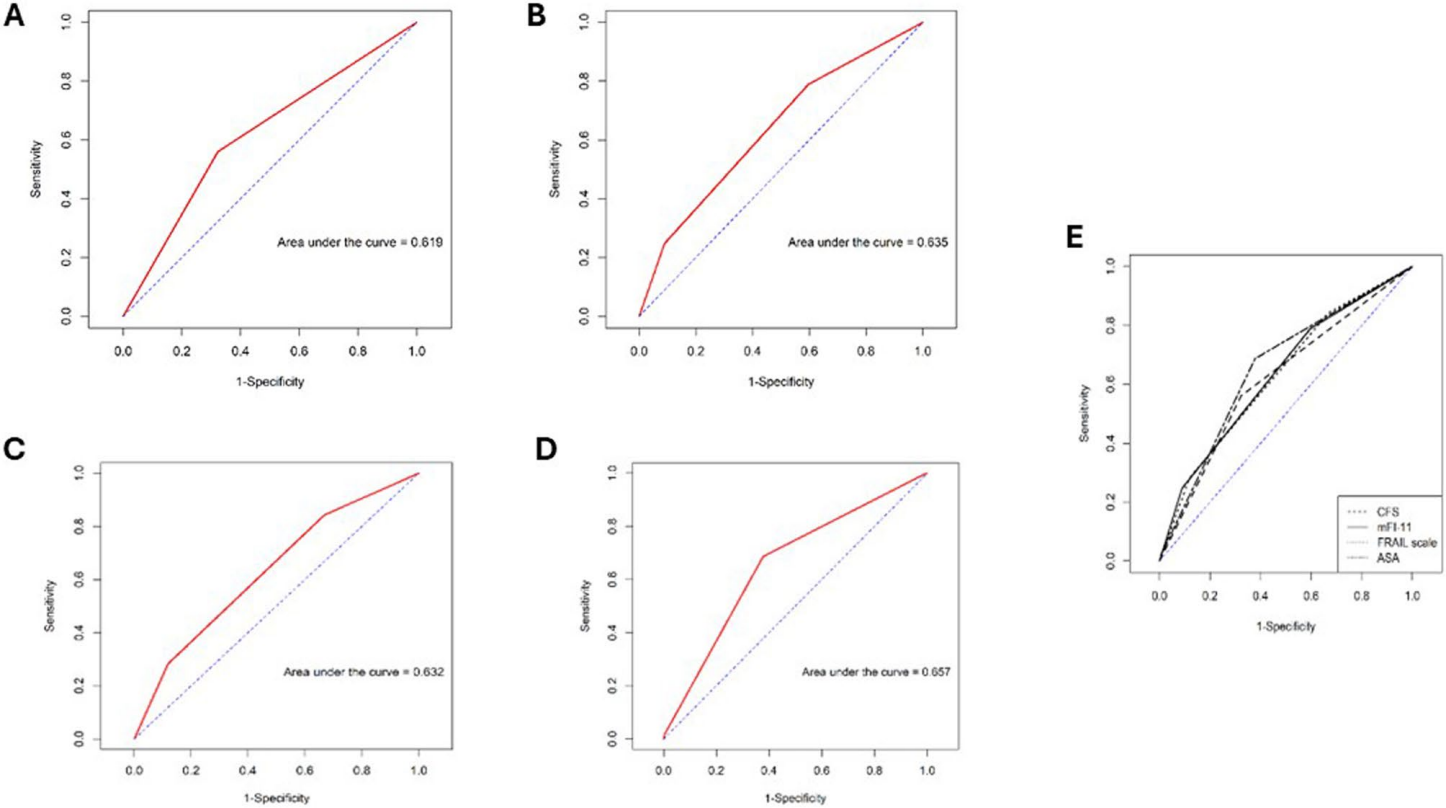
Receiver operating characteristic (ROC) analysis of the CSF(**A**), mFI-11 (**B**), FRAIL scale (**C**), ASA classification (**D**), and all combined (**E**) in predicting postoperative complications, according to Clavien–Dindo classification ≥ 2

**Fig. 3:**
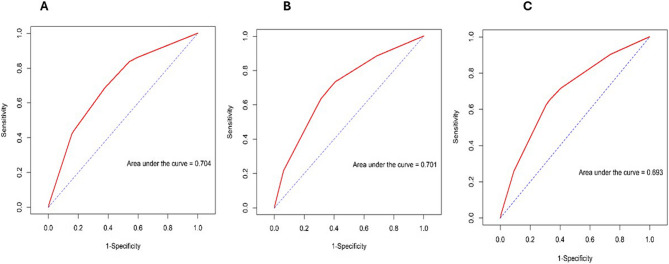
Receiver operating characteristic (ROC) analysis of combined ASA classification and CSF (**A**), combined ASA classification and mFI-11 (**B**), and combined ASA classification and FRAIL scale (**C**) in predicting postoperative complications, according to Clavien–Dindo classification ≥ 2

The mFI-11 had the highest AUC at 0.635 (95% CI: 0.5902–0.6794), followed by the FRAIL scale at 0.632 (95% CI: 0.5881–0.6756), and the CFS at 0.619 (95% CI: 0.5742–0.6637) (Figure 2). The AUC of the ASA classification was 0.657 (95% CI: 0.6152–0.6988) (Figure 2). The predictive ability of the ASA classification was similar to that of the frailty assessment tools. Differences were observed between the ASA classification and the CFS (p = 0.1904), mFI-11 (p = 0.4058), and FRAIL scale (p = 0.3099). When the ASA classification was combined with each frailty scale, the predictive ability improved. Specifically, the AUC increased to 0.704 (95% CI: 0.6595–0.7479), 0.701 (95% CI: 0.656–0.746), and 0.693 (95% CI: 0.6476–0.7383) for the CFS, mFI-11, and FRAIL scale, respectively (Figures 3). The differences in predictive ability among the frailty scales were not significant. Nonetheless, significant differences were observed between when the ASA classification was combined with each frailty scale: ASA+CFS (p < 0.001), ASA+mFI-11 (p < 0.001), and ASA+FRAIL scale (p = 0.0015).

## Discussion

Our study demonstrated that frailty, as measured using the CFS, mFI-11, and FRAIL scale, was common among older Thai patients undergoing intermediate- to high-risk non-cardiac surgery. Frailty prevalence varied across the different tools, consistent with the findings of studies showing that instrument choice influences frailty estimates [18, 30], reflecting differences in how each tool defines and measures frailty and underscoring the need for context-specific selection in clinical practice. [8, 18, 30–32].

### Association between frailty and postoperative complications

Frail patients were more often classified as having higher Clavien–Dindo grades, reflecting more severe postoperative complications. Overall, postoperative morbidity was significantly higher in frail than non-frail patients across all frailty assessment tools, consistent with the findings of previous studies showing frailty as a predictor of adverse surgical outcomes [8, 21, 32–34]. In multivariable models, only the CFS remained an independent predictor, which may be explained by the broader scope of the CFS, which incorporates both functional ability and mental status, providing a more comprehensive assessment of patient vulnerability. By comparison, the FRAIL scale focuses primarily on physical performance, while the mFI-11 emphasizes comorbidities and activity limitations. Prior studies similarly reported the CFS as a strong predictor of complications in older surgical patients [35]. However, some investigations have demonstrated significant associations with the mFI-11 [21, 36], while others found no predictive value for any frailty tool [30]. Overall, these findings highlight that while frailty is consistently linked to poorer outcomes, the predictive accuracy varies depending on the assessment instrument used [8, 21, 30, 32–36].

### Specific postoperative outcomes and frailty

Frailty identified by the CFS was associated with a greater need for blood transfusions and more frequent unplanned returns to the operating room. Interestingly, frail patients exhibited a lower incidence of cardiac complications than non-frail patients. This unexpected finding may reflect selection bias, as the frailest patients with severe cardiac disease are not offered surgery or are managed more conservatively.

Additionally, frailty was consistently linked to prolonged hospital stay across all assessment tools, indicating increased healthcare resource utilization. Similarly, previous studies have shown that frailty indices predict longer LOS and higher hospital costs [37–39].

### Frailty and 30-day mortality

Regarding mortality, the mFI-11 exhibited a stronger association with 30-day mortality than did the CFS or FRAIL scale, likely due to its inclusion of comorbidities that are closely tied to short-term survival. This pattern is consistent with the findings of other studies reporting higher 30-day mortality among patients classified as frail by the mFI-11 [36, 40]. The discrepancy may reflect the broader scope of the mFI-11, which incorporates comorbidities and functional status, thereby providing a more comprehensive risk profile. Conversely, Narula et al. [41] demonstrated that frailty assessed with the CFS was significantly associated with 30-day mortality; other investigators have found similar associations using the FRAIL scale [42]. This suggests that different tools may have complementary strengths depending on the outcome of interest [36, 40–42].

### Predictive ability of the frailty tools for postoperative complications

Regarding predictive performance, all three frailty measures exhibited moderate ability to discriminate postoperative complications. While none clearly outperformed the ASA classification alone, combining frailty assessments with ASA improved overall risk prediction, which is consistent with the results of prior studies suggesting that frailty provides complementary prognostic information and should be incorporated into existing risk assessment models [18, 30, 43, 44].

### Strengths and limitations

The strengths of this study include its prospective design, large sample size, and the use of multiple validated frailty instruments. Nonetheless, its single-center setting limits generalizability, and the observational design introduces potential biases such as selection bias, information bias, and residual confounding. Additionally, only short-term (30-day) outcomes were assessed, and long-term frailty consequences were not captured.

## Conclusion

Frailty is a significant predictor of postoperative complications in older patients undergoing non-cardiac surgery. The CFS, mFI-11, and FRAIL scale are feasible tools for assessing frailty in this patient population. When combined with the ASA classification, they enhanced predictive accuracy, with the CFS demonstrating a superior predictive ability for postoperative complications. Incorporating frailty assessments into routine preoperative evaluations can improve patient outcomes by identifying those at a higher risk of adverse events and tailoring perioperative care accordingly. Future studies should focus on refining frailty assessment methods and exploring interventions to mitigate the impact of frailty on surgical outcomes.

## Data Availability

All data generated or analyzed during this study are included in this published article.

## Abbreviations

CFS: Clinical Frailty Scale
mFI-11: Modified Frailty Index-11
ASA: American Society of Anesthesiologists
CI: Confidence interval
OR: Odds ratio
AUC: Area under the receiver operating characteristic curve
ROC: Receiver operating characteristic
LOS: Length of hospital stay
